# A randomized trial to assess the impact of opinion leader endorsed evidence summaries on the use of secondary prevention strategies in patients with coronary artery disease: the ESP-CAD trial protocol [NCT00175240]

**DOI:** 10.1186/1748-5908-1-11

**Published:** 2006-05-06

**Authors:** Finlay A McAlister, Miriam Fradette, Michelle Graham, Sumit R Majumdar, William A Ghali, Randall Williams, Ross T Tsuyuki, James McMeekin, Jeremy Grimshaw, Merril L Knudtson

**Affiliations:** 1 The Department of Medicine, University of Alberta, Edmonton, Canada; 2 The Epidemiology Coordinating and Research (EPICORE) Centre, University of Alberta, Canada; 3 The Department of Medicine, University of Calgary, Calgary, Canada; 4 The Royal Alexandra Hospital, Edmonton, Canada; 5 The University of Ottawa Health Research Unit, Ottawa, Canada

## Abstract

**Background:**

Although numerous therapies have been shown to be beneficial in the prevention of myocardial infarction and/or death in patients with coronary disease, these therapies are under-used and this gap contributes to sub-optimal patient outcomes. To increase the uptake of proven efficacious therapies in patients with coronary disease, we designed a multifaceted quality improvement intervention employing patient-specific reminders delivered at the point-of-care, with one-page treatment guidelines endorsed by local opinion leaders ("Local Opinion Leader Statement"). This trial is designed to evaluate the impact of these Local Opinion Leader Statements on the practices of primary care physicians caring for patients with coronary disease. In order to isolate the effects of the messenger (the local opinion leader) from the message, we will also test an identical quality improvement intervention that is not signed by a local opinion leader ("Unsigned Evidence Statement") in this trial.

**Methods:**

Randomized trial testing three different interventions in patients with coronary disease: (1) usual care versus (2) Local Opinion Leader Statement versus (3) Unsigned Evidence Statement. Patients diagnosed with coronary artery disease after cardiac catheterization (but without acute coronary syndromes) will be randomly allocated to one of the three interventions by cluster randomization (at the level of their primary care physician), if they are not on optimal statin therapy at baseline. The primary outcome is the proportion of patients demonstrating improvement in their statin management in the first six months post-catheterization. Secondary outcomes include examinations of the use of ACE inhibitors, anti-platelet agents, beta-blockers, non-statin lipid lowering drugs, and provision of smoking cessation advice in the first six months post-catheterization in the three treatment arms. Although randomization will be clustered at the level of the primary care physician, the design effect is anticipated to be negligible and the unit of analysis will be the patient.

**Discussion:**

If either the Local Opinion Leader Statement or the Unsigned Evidence Statement improves secondary prevention in patients with coronary disease, they can be easily modified and applied in other communities and for other target conditions.

## Background and rationale

Coronary artery disease (CAD) leads to substantial morbidity and mortality. Control of the CAD epidemic will require a multifaceted strategy including primary prevention maneuvers – some designed for the general population and some targeting only high-risk individuals, and secondary prevention maneuvers targeted at those with established disease. Many of the risk factors for CAD are modifiable and improving these risk factors has been shown to reduce the subsequent occurrence of myocardial infarction (MI) or death in patients with CAD. In particular, there is strong evidence supporting the following five therapies or maneuvers for secondary prevention in patients with CAD: statins (cholesterol lowering drugs), smoking cessation, antiplatelet agents, beta-blockers, and ACE (angiotensin converting enzyme) inhibitors.

### Statins

Large-scale epidemiologic studies have shown there is a strong, consistent and graded relationship between cholesterol levels and mortality from CAD [1]. A series of 11 randomized trials (Table 1) [2-12] over the past decade have confirmed that initiating statin therapy in patients with CAD reduces the occurrence of vascular events; indeed, the relative risk reductions appear to be independent of baseline cholesterol levels, at least in the range of cholesterols tested in the trials. Two other large trials [13,14] targeted patients for primary prevention of MI and, although they may well have included some patients with occult CAD, are not included in Table 1. The only large statin trial that failed to demonstrate a statistically significant benefit with statin use (ALLHAT-LLT) was likely contaminated by very high rates of statin use in the "control" arm of that trial[15]. A meta-analysis of these trials confirmed that statins are clearly beneficial for secondary prevention in all subgroups of CAD patients, including those with LDL cholesterol levels ≤ 2.5 mmol/L and those without prior MI[16].

**Table 1:** Features of randomized statin secondary prevention trials designed to detect differences in clinically important end-points

**Trial**	**Treatment (mg/day) and Follow-up Duration**	**Key Eligibility Criteria**	**Number of Patients**	**Mean Age (yrs)**	**% Change in LDL-c**	**Relative Risk Reduction, Mortality and MI (95% CI)**
**4S [2]**	Simvastatin 20 mg for 5.4 yrs (median)	35–70 yrs, prior angina or AMI, fasting total cholesterol 5.5–8.0 mmol/L	4444	58.6	-35%	30% (15% to 42%) and 27% (20% to 34%)
**LIPID [3]**	Pravastatin 40 mg for 6.1 yrs (mean)	31–75 yrs, prior AMI or unstable angina, fasting total cholesterol 4 – 7 mmol/L	9014	62	-25%	22% (13% to 31%) and 29% (18% to 38%)
**CARE [4]**	Pravastatin 40 mg for 5.0 yrs (median)	21–75 yrs, prior AMI, fasting LDL cholesterol 3.0–4.5 mmol/L	4159	59	-28%	9% (-12% to 26%) and 25% (8% to 39%)
**MRC/BHF Heart Protection Study[5]**	Simvastatin 40 mg for 5.0 yrs (mean)	40–80 yrs, increased risk of CV death (due to known atherosclerotic disease, or diabetes, or hypertension with other CV risks)	20 536	NR	-29%	13% (6% to 19%) and 27% (21% to 33%)
**MIRACL [6]**	Atorvastatin 80 mg for 16 weeks (mean)	18 – 77 yrs, ACS, screening cholesterol <7.0 mmol	3086	65	-52%	6% (-31% to 33%) and 10% (-16% to 31)
**LIPS [7]**	Fluvastatin 80 mg for 3.9 yrs (median)	18 – 80 yrs, after percutaneous intervention, screening cholesterol 3.5–7.0 mmol	1677	60	-27%	31% (17% to -14%) and 19% (62% to -24%)
**PROSPER[8]**	Pravastatin 40 mg for 3.2 yrs (mean)	70–82 yrs, with vascular disease or at high risk, screening cholesterol 4.0–9.0 mmol/L	5804	75	-34%	3% (17% to -14%) and 14% (-3% to +28%)^1 ^
**ASCOT [9]**	Atorvastatin 10 mg for 3.3 yrs (median)	40–79 yrs, hypertension plus >3 other cardiovascular risk factors, screening cholesterol ≤ 6.5 mmol/L	10 305	63	-35%	13% (29% to -6%) and 36% (17% to 50%)
**PROVE IT-TIMI 22 [10]**	Atorvastatin 80 mg vs. Pravastatin 40 mg for 2.0 yrs (mean)	> 18 yrs, ACS, screening cholesterol ≤ 6.21 mmol/L or 5.18 mmol/L if on lipid lowering therapy	4162	58.3	Atorva: -42% Prava: -10%	28% (-2% to +50%) and 13% (-8% to 32%)^1 ^
**TNT [11]**	Atorvastatin 80 mg vs. Atorvastatin 10 mg for 4.9 yrs (median)	35–75 yrs, stable CAD, LDL-c < 3.4 mmol/L	10 001	61	Atorva 80 mg: -21% Atorva 20 mg: no change	-1% (-19% to +15%) and 22% (7% to 34%)^1 ^
**IDEAL [12]**	Atorvastatin 80 mg vs. Simvastatin 20 mg for 4.8 yrs (median)	18–80 years, prior AMI	8888	62	Atorva : -34% Simva : -17%	2% (-13% to 15%) and 17% (2% to 29%)^1 ^
**ALLHAT-LLT [15]**	Pravastatin 40 mg for 4.8 yrs (mean)	Hypertension, older than 55 years, at least one other cardiac risk factor and LDL-c 3.1–4.9 mmol/L without known CAD or 2.6–3.3 with known CAD	10 355	66	-17%	1% (-11% to +11%) and 9% (-4% to +21%)

1. Based on hazard ratio; LDL-c= LDL cholesterol; CAD = coronary artery disease; AMI = acute myocardial infarction; ACS = acute coronary syndrome; CV = cardiovascular; Atorva = atorvastatin; Prava = Pravastatin; Simva = simvastatin.

### Smoking cessation

Cigarette smokers with CAD are at increased risk for MI – relative risks range from 1.4 to 2.2 in cohort studies[1]. There is evidence that smoking cessation lowers the risk of recurrent myocardial infarction by almost 50% within 2 years,[17] and systematic reviews have shown that one-time advice from physicians during routine office visits increases the annual rate of smoking cessation by 2%. Interventions such as bupropion and/or nicotine replacement therapies may also increase cessation rates. [18-20] Patients with symptomatic CAD may be even more receptive to smoking cessation advice, with up to one-third quitting smoking after acute MI[21].

### Antiplatelet agents

The Antithrombotic Trialists' Collaboration[22] included 27 trials in 39,308 patients with a history of MI: meta-analysis of the data confirmed that aspirin conferred a 23% relative reduction in subsequent rates of MI, stroke, or vascular death. This systematic review also included 53 trials in 17,394 patients with CAD but no prior MI: the relative risk reduction with aspirin was 30% for vascular events.

### Beta-blockers

A systematic review of 55 trials in MI survivors demonstrated a convincing survival benefit with beta-blockers (RRR 25%), irrespective of baseline clinical risk factors[23,24]. Although beta-blockers have not been shown to be more efficacious than long-acting calcium-channel blockers or nitrates in those CAD patients without a history of MI (a systematic review of 90 comparative trials)[25], there is some data suggesting that beta-blockers reduce cardiac morbidity and mortality in CAD patients without prior MI. [26-31] Guidelines from the American College of Physicians, the American Heart Association, and the American College of Cardiology recommend the use of beta-blockers as first-line therapy for angina in patients without contraindications[32]. Thus, a case can be made for recommending beta-blockers in all CAD patients who have already suffered a MI or who are symptomatic, unless contraindicated.

### ACE inhibitors

The Heart Outcomes Prevention Evaluation (HOPE) trial showed that compared to placebo, ramipril reduced cardiovascular events (nonfatal MI, stroke, or vascular death) by 22% in high-risk patients 55 years or older with evidence of vascular disease or diabetes, plus one other cardiovascular risk factor[33]. The relative efficacy was similar in the 7477 patients with known CAD, irrespective of whether they had already suffered a MI or not. Similar benefits with ACE inhibition in CAD patients were seen in the EURopean trial On Reduction of cardiac events with Perindopril in stable coronary Artery disease (EUROPA)[34] and the Simvastatin/Enalapril Coronary Atherosclerosis Trial (SCAT)[35]. Although the placebo-controlled PEACE (Prevention of Events with Angiotensin-Converting Enzyme Inhibitor) trial [36] did not find a reduction in cardiovascular morbidity or mortality with trandolapril, participants' cardiovascular risk factors were so well controlled at baseline in the PEACE trial that the event rates in the placebo group were far lower than in the HOPE, EUROPA, or SCAT studies – and were sufficiently low enough so that the study was under-powered to detect a significant benefit in the ACE inhibitor arm. Thus, it seems reasonable to recommend that ACE inhibitors be strongly considered for patients with CAD and without contraindications, particularly those with suboptimal control of risk factors such as LDL (low-density lipoprotein) cholesterol – the very patients of interest in the ESP-CAD trial described below.

#### Coronary artery disease: missed opportunities for secondary prevention

Despite the abundant evidence base for secondary prevention, practice audits consistently demonstrate substantial "care gaps" between this evidence and clinical reality, in that many patients with CAD are not offered all possible opportunities for the secondary prevention of MI or death (see Table 2). For example, even after an acute MI, almost one-fifth of patients with CAD continue to smoke, more than half with hypertension or hyperlipidemia have poorly controlled blood pressures or lipid levels, and proven efficacious therapies such as antiplatelet agents, beta-blockers, ACE inhibitors, and statins are under-prescribed. [37-50] Even those patients that receive therapies (i.e. statins) rarely achieve the recommended target levels for the treated risk factor, either due to suboptimal dose titration or poor patient adherence (statin persistence has been shown to range from 43% to 75% at one year). [51-53] Importantly, these "care gaps" are linked to poor patient outcomes, and closure of these care gaps improves prognosis[39,54]. Clearly, a means to help translate this evidence into clinical practice is urgently required.

**Table 2:** Provision of proven efficacious therapies in patients with CAD, multi-centre studies since 1995

**Setting (ref)**	**Sample Size**	**Statin Use**	**ACE Inhibitor Use**	**Beta- blocker Use**	**Antiplatelet Use**	**Current Smokers**	% with cholesterol at or below target*
***Audits from General Practices:***							
Canada (42)	4315	38%	NR	NR	53%	25%	14%
Canada (43)	NR	NR	NR	NR	54%	NR	NR
USA (46)	11 745	NR	NR	21%	NR	NR	NR
UK (41)	1921	NR	10%	32%	63%	18%	17%
UK (44)	24 431	16%	13%	22%	50%	24%	56%
Canada (49)	3721	100%	NR	NR	NR	17%	73%
International REACH Registry (50)	40 258	76%	51%	63%	86%	13%	NR
***Audits in patients discharged after acute myocardial infarction or coronary artery bypass surgery:***							
USA (39)	201 752	NR	30%	34%	83%	NR	NR
USA (37)	1710	12%	NR	44%	53%	NR	NR
USA (38)	622	37%	NR	23%	46%	25%	15%
USA (45)	190 015	NR	31%	NR	NR	NR	NR
USA (47)	25 000	NR	NR	NR	81%	NR	NR
Europe (40)	3379	58%	43%	66%	84%	21%	41%
Ontario(48)	9667	40%	65%	68%	NR	NR	NR
Quebec(48)	4790	43%	57%	68%	NR	NR	NR
British Columbia(48)	2570	42%	58%	61%	NR	NR	NR
Nova Scotia(48)	761	36%	58%	83%	NR	NR	NR
Alberta (APPROACH patients)**	5104	34%	39%	61%	81%	NR	NR

Note that while the general practice audits represent cross-sectional data at varying times after the diagnosis of CAD, the audits in patients discharged after acute MI generally represent prescribing data between 90 days and 120 days after MI.

* Target cholesterol defined as LDL cholesterol ≤ 2.6 mmol/L or total cholesterol ≤ 5.0 mmol/L.

** Medications in use at the time of the cardiac catheterization (no data available on prescriptions in follow-up). [Colleen Norris, personal communication, August 2003]

#### Current strategies to close care gaps are often ineffective

In examining why care gaps are present, it has consistently been shown that multiple barriers (patient-, physician-, and health care system-related) are often responsible for the lack of implementation of proven efficacious therapies and traditional means of educating practitioners (journal articles, continuing medical education conferences, grand rounds lectures, *et cetera*) are usually ineffective in altering practice[55,56]. Disease management programs employing specialized clinics or multidisciplinary teams have been shown to improve the management of patients with CAD; however, these programs are often difficult to implement on a widespread scale and are only available for a minority of eligible patients[57]. Simpler, cheaper, and more effective means of increasing the prescribing of proven therapies for CAD patients are needed.

#### The potential role of point-of-care reminders and opinion leaders in closing care gaps

In looking for a simple, effective and evidence-based means to improve the quality of care for patients with CAD, we have isolated 2 key elements – point-of-care reminders and educational materials endorsed by local opinion leaders that form the basis of the quality improvement intervention to be tested in this trial[58].

The interface between hospital-based specialists and community-based primary care physicians is frequently imperfect, and this system barrier likely affects the quality of care for patients with CAD. Reminder systems, particularly those which are clear, patient-specific, and delivered at the point of care, can improve communication and the delivery of health services in numerous settings, although the effects are often modest[59]. A recent systematic review, however, concluded that the effects of point-of-care reminders sent by specialists to primary care physicians had been inadequately evaluated[59].

This is particularly significant because surveys of primary care physicians consistently confirm the importance of colleagues and local consultants on patterns of practice[60,61]. In fact, several recent studies suggest that the mere provision of evidence without specialist input, even with a point-of-care reminder at the time patients are being seen, may not be enough to change practice in CAD. For example, although the mailing of patient-specific reminders (from the local health authority) about secondary prevention therapies to the primary care physicians of MI survivors in England led to higher rates of cholesterol measurement and recording of cardiac risk factors in these patients, there was no appreciable difference in statin prescribing rates[62]. Similarly, faxing care management summary sheets (listing diagnoses, medications, pertinent laboratory data, and guideline recommendations for each patient) to the primary care physicians of patients with diabetes and dyslipidemia did not significantly impact statin prescription rates[63]. Finally, four trials testing the effects of computerized decision support systems that prompted primary care practitioners with reminders and management guidelines (which were not explicitly endorsed by local opinion leaders) when they were seeing patients with CAD reported negligible improvements in prescribing of statins or other proven efficacious therapies compared to controls. [64-67]

Local opinion leaders are well-known, respected health care professionals who are trusted by their peers to evaluate medical innovations within the local context. [68-70]. Since they influence patterns of practice in the community, their participation in any program of quality improvement is essential. Yet, the use of local opinion leaders to influence physician practice has only been tested in 10 randomized trials[69,71,72]. While eight of the nine trials that measured practice patterns showed some improvements with opinion leaders, only three demonstrated statistically significant benefits. [72-74] All three of these trials assessed labour-intensive, expensive, hospital-based educational interventions spearheaded by a small number of opinion leaders for inpatient conditions (delivery by cesarean section, treatment of acute MI, and treatment of unstable angina). A tenth trial evaluated the impact of a multifaceted intervention that included physician and nurse opinion leaders (as two of the 10 factors included in the intervention) on management of patients with acute MI. They demonstrated improvements in some of their quality indicators, however, it is impossible to gauge the efficacy of opinion leaders alone in this study[54]. Although the use of local opinion leaders to influence the outpatient management of common conditions (such as CAD) holds great promise, as of yet this is a promise unfulfilled, and a hypothesis that needs to be tested.

#### Interventions must be practical to influence community-based practice

When testing the effect of local opinion leaders in the outpatient setting, the generalizability of the intervention is of utmost concern. Thus, the focus must be on a practical means of incorporating the influence of these individuals into everyday practice. Previous studies have established that the information format favoured most by front-line clinicians is a one-page summary of guidelines or evidence[60,75]. Moreover, two studies have established that specific guidelines are substantially more effective in influencing physician behaviour than non-specific guidelines[76,77]. Finally, there is speculation that providing objective proof of disease (e.g., a coronary angiogram report), along with the evidence, may enhance the impact of the evidence with physicians. However, whether such a picture really is worth a 1,000 words has never been rigorously evaluated in a randomized trial.

#### Work preceding this trial

With these considerations in mind, we surveyed all primary care physicians in Edmonton and Calgary, Canada and asked them to nominate local opinion leaders for CAD in each region, using a previously validated sociometric survey tool as described fully elsewhere[68,78]. These local opinion leaders agreed to participate in this project and worked in concert with clinical epidemiologists to generate and endorse one-page evidence summaries and treatment recommendations for the management of patients with CAD (hereafter referred to as the "Local Opinion Leader Statement"**- **see Additional file 1). We chose to emphasize statins in the Local Opinion Leader Statement because we felt the evidence base for statins in patients with CAD was robust and applicable to virtually all patients with CAD (see Table 1). The recommendations will be distributed to the primary care physician by fax, along with patient-specific coronary angiogram results. This will act as both a source of credible and convincing information and a specific reminder for action at the next patient encounter.

Local opinion leaders are not always self-evident and conducting surveys to identify them for each condition and in each locale would be difficult. Thus, it is important to be certain that any benefits seen with a local opinion leader-based intervention are truly due to the local opinion leader (the messenger) and not just the message. For that reason, we have included a third arm in our trial which will involve exposure to a quality improvement initiative that is identical in every respect to the local opinion leader statement – but without the local opinion leader signature (hereafter referred to as "Unsigned Evidence Statement" – see Additional file 2). Of note, this arm of the study will essentially duplicate the typical point-of-care reminder studies discussed earlier. [63-67]

#### Aim of the study

This trial is designed to test two interventions for improving the quality of care for patients with established CAD. The principal hypothesis to be tested is: Does a local opinion leader-based quality improvement intervention influence primary care physicians to increase the provision of secondary prevention therapies in their patients with known CAD compared to usual care? The secondary hypotheses to be tested are: (1) Does the same quality improvement intervention, but without explicit local opinion leader endorsement (i.e., without the local opinion leaders' signatures), improve the provision of secondary prevention maneuvers in CAD patients compared to usual care? And, (2) does local opinion leader endorsement increase the effectiveness of the quality improvement intervention?

## Methods

### Study design

**Figure 1:**
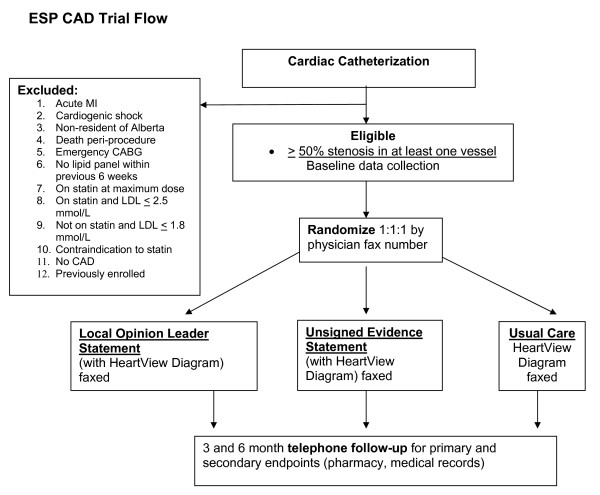
Trial Flow Proposed patient enrollment and randomization procedures.

The study design is a randomized clinical trial testing three different intervention policies: Usual care versus Local Opinion Leader Statement versus Unsigned Evidence Statement (see Trial Flow in Figure 1). The target population is patients with CAD proven on coronary angiography. As there is a potential for a physician or a group practice of physicians to have patients randomized to more than one arm of the study, cluster randomization at the level of the practice will be employed to avoid contamination. [79] Thus, this represents the optimal design for evaluating quality improvement interventions[80].

### Details of the intervention

The Local Opinion Leader Statement is a one-page summary of evidence-based secondary prevention strategies and treatment recommendations for patients with CAD, and contains the signatures of all five CAD local opinion leaders identified by our survey of primary care practitioners (see Additional file 1). The emphasis is on statin prescribing – as a result, statins are recommended first (listing only those statins which have been proven efficacious in large trials), and the letter explicitly mentions their starting and target doses, usual titration schedules, and monitoring parameters. For each of the other secondary prevention therapies (ACE inhibitors, antiplatelet agents, beta-blockers), we list only the drug class in the Opinion Leader Statement without explicit statements about dosing, titration, or monitoring parameters.

The Local Opinion Leader Statement will be imprinted with the name of the patient and addressed directly to the primary care physician. The statement will be faxed to the physician following the completion of the patient's coronary angiogram along with objective evidence of the patient's CAD (the Alberta Provincial Project for Outcome Assessment in Coronary Heart Disease [APPROACH] HeartView Diagram which documents the extent of the patient's coronary atherosclerosis)[81]. All of the faxes (APPROACH HeartView diagrams and the one page statement) will be generated and sent automatically using a software program that has been developed for this trial and embedded in the APPROACH software. It is intended that the statement and the HeartView Diagram will become part of the patient's medical record and will serve as a reminder for action at the next patient visit.

The Unsigned Evidence Statement, identical to the Local Opinion Leader Statement in content but without the local opinion leader signatures (see Additional file 2), will be faxed to the primary care physician, along with the APPROACH HeartView Diagram, in the same manner as described above.

Physicians of control patients (usual care) will receive a fax containing only the APPROACH Heartview Diagram. This will ensure that all physicians and patients receive the same number of study related materials and encounters, with the only difference being the content of the fax.

### Study setting

All three cardiac catheterization laboratories in the province of Alberta, Canada (total population 3.1 million people) are participating in this trial.

### Study participants

Eligible patients (and their primary care physicians) will be identified by the research assistants at the time of their cardiac catheterization and approached for written informed consent to participate in the study. Specific criteria for inclusion and exclusion include the following:

#### Inclusion criteria

Alberta residents older than age 18 who undergo a cardiac catheterization and are diagnosed with CAD on the basis of coronary angiography, demonstrating a stenosis in at least one coronary vessel of ≥ 50%[82].

#### Exclusion criteria

Patients will be excluded if: (1) they have not had a fasting lipid panel done in the six weeks prior to their cardiac catheterization; (2) they are already on a statin at maximal dose; (3) they are on a statin/lipid-lowering drug and the LDL cholesterol level is ≤ 2.5 mmol/l; (4) they are not on a statin but their fasting LDL is ≤ 1.8 mmol/L; (5) they do not have CAD proven on catheterization; (6) they are undergoing catheterization in the setting of an acute coronary syndrome or cardiogenic shock; (7) they die during the catheterization or require emergency bypass surgery; (8) the catheterization is being done as part of a research protocol; (9) they do not have an identifiable primary care physician; (10) they have contraindications to statin use (history of cirrhosis or inflammatory muscle disease, serum creatinine ≥ 200 umol/L, women of child-bearing age, or prior allergy to statins); and/or (11) they are already enrolled in ESP-CAD.

### Allocation to experimental arms

Randomization will take place 1:1:1 at the level of the practice (either individual physician for solo practitioners or clinic for those physicians who practice in a group setting) following the completion of the patient's coronary angiogram using a 'real-time' central randomization system with allocation concealment embedded in the APPROACH software. After a practice's first patient is randomized, all subsequent patients from that practice will be assigned to whatever treatment arm the first patient was randomized to.

While this is a form of cluster randomization, we anticipate the design effect to be negligible since the majority of physicians will contribute no more than one or two study patients, and thus all sample size and analytic considerations use the patient as the unit of analysis and the unit of causal inference.

### Outcome measures

We decided to focus on only those secondary prevention maneuvers that have strong evidence of survival benefits and are readily measurable from patient self-report and/or examination of pharmacy records. We decided not to examine factors (such as blood pressure control or LDL cholesterol levels) that would require in-person patient assessments, as this would substantially increase the complexity and expense of this study. If the interventions tested in this study are effective, then subsequent studies will extend the interventions (and their assessment) to address other cardiovascular risk factors such as control of blood pressure.

Although we mention five secondary prevention maneuvers in the local opinion leader and unsigned evidence statements (see Additional Files 1 and 2), we chose to emphasize statin prescribing in the statements (and as our primary outcome measure) because we felt the evidence for using statins in all patients with CAD (regardless of baseline cholesterol level) is more robust than the evidence for the use of ACE inhibitors or beta-blockers in all patients. Although the evidence in support of antiplatelet agents also is robust, we chose not to make this the primary outcome because of the likelihood that ASA prescribing may be close to maximal already (see Table 2).

#### Primary outcome

The primary outcome measure is a composite measure representing improvement in statin-related secondary prevention consisting of: 1) provision of a statin sample, or 2) provision of a statin prescription, or 3) increasing the dosage of a statin within the first six months post-angiogram. As this is a composite end-point, only the first event attained in the cluster will be counted for analysis, although all events will be recorded in the database.

Six months was chosen for the primary and secondary outcomes because the average number of physician visits in our audit of trial-eligible patients attending the University of Alberta cardiac catheterization laboratory in the 2003 year was 1.8 visits in six months. As we are assessing the impact of the Local Opinion Leader Statement and Unsigned Evidence Statement on physician practice patterns, for the purposes of this study we are interested solely in evaluating attempted practice change. In other words, if a patient is provided with a statin sample or a statin prescription, or the dosage is increased by any of the patient's physicians, it would still count as a positive outcome for the main study analysis, even if the patient cannot tolerate the medication and discontinues it or is noncompliant during follow-up. We recognize that patient (and physician) long-term compliance with preventive therapies is important, and will collect data on persistence rates with secondary prevention maneuvers over the six months of the study. We anticipate that this data will serve as pilot data for a planned future study on interventions to improve provider/patient persistence rates.

We will examine the primary outcome rates in all three treatment arms as follows: the primary comparison will be between those patients randomized to the Local Opinion Leader Statement versus usual care, and the secondary comparisons will be (a) between those patients randomized to the Unsigned Evidence Statement versus usual care, and (b) between those patients randomized to the Local Opinion Leader Statements versus Unsigned Evidence Statements.

#### Secondary outcomes

Secondary outcomes will include the provision of other proven efficacious medications for CAD by six months, including ACE inhibitors, beta-blockers, and antiplatelet agents; these medications will be considered independently as individual end-points in eligible patients. Moreover, we will examine changes in the provision of other lipid-lowering medications (fibrates, niacin, and/or resins), as well as self-reported smoking rates, receipt of smoking cessation advice, provision of nicotine replacement products, or buproprion in trial patients within six months of their angiogram. We also will record whether study participants have a fasting lipid profile done in the six months post-angiogram and, for the subgroup of patients who have a fasting lipid profile done during follow-up (i.e., done for clinical reasons by their attending physicians, not mandated by the trial protocol), we will examine the proportion of patients achieving target LDLs compared to baseline. Finally, we also will analyze clinical events (MI, stroke, admissions for CAD, total hospitalizations, and mortality) in the three arms of this study, although the study is not powered to find any differences in these end-points over such a short timeframe.

### Study procedures and data collection

Baseline data (including demographics, comorbidities, medication use, cholesterol levels, and sociodemographic variables) will be collected by research personnel using a standardized abstraction instrument at the time of the patient's catheterization. The primary source of outcome data for the study will be patient self-report on telephone contact at three and six months, with cross-referencing to pharmacy records for medications, centralized laboratory data for fasting lipid panels, and medical records for clinical outcomes. In an earlier study, we found a high degree of agreement between patient self-report of statin and ACE inhibitor use and data from dispensing pharmacies (simple agreements ranged between 88% to 99% and kappas were 0.78 to 0.93)[83]. Vital status will be queried via the APPROACH database. The outcome data will be abstracted using standardized forms in a secure database housed in the Epidemiology Coordinating and Research (EPICORE) Centre, University of Alberta, Canada.

Investigators, outcome assessors, and study patients will be masked to allocation status. Primary care physicians cannot be blinded to allocation status. Follow-up data will be collected without knowledge of allocation status in an independent and blinded fashion, and statistical analyses will be conducted by a statistician blinded to allocation status.

### Sample size

In a survey of 22 members of the divisions of cardiology and general internal medicine at the University of Alberta, we determined that the "minimal" clinically important difference for this particular intervention to be considered useful was a 15% absolute improvement over and above usual care. After six months, we estimate that no more than 20% of control patients will have attained our composite primary outcome, given that patients who are already on a statin and have optimal LDL cholesterol levels will be excluded from this study at baseline. We calculated our sample size to detect a 15% absolute increase in the primary outcome, set the α error rate at 0.05 (2-sided), and the β error at 0.20 (power 80%) – this yielded a sample size of 138 patients per study arm. Allowing for losses during follow-up, the ability to examine each of the conditions separately, and the possibility of a very small design effect associated with patient clustering, the total sample size has been adjusted upwards to 160 patients per arm (480 in total).

### Statistical analyses

Intention-to-treat analyses will be carried out with patients as the unit of analysis. Although the physician will be the unit of allocation, we anticipate a very small design effect, and the outcomes for individual patients will be clinically and statistically independent of each other.

The primary outcome will first be tested using the chi square statistic to compare the attainment of our primary statin outcome within six months in patients randomized to the Local Opinion Leader Statement versus patients randomized to the Unsigned Evidence Statement. If the impact of the Local Opinion Leader Statement is significantly different from that of the Unsigned Evidence Statement, we will compare each to usual care separately. If the impact of the Local Opinion Leader Statement and the Unsigned Evidence Statement are similar, we will combine data from both arms and compare this with usual care, which will essentially be a test of point-of-care reminders versus usual care.

In order to investigate what factors are associated with changes in the primary outcome (our dependent binary variable), and to control for the possibility of potential imbalances in patient-level characteristics at baseline, multivariable logistic regression analyses will be used to examine those variables that are deemed to be clinically important (i.e., age, gender) or that differ statistically at a p-value < 0.10 between study arms. In addition, to examine the possibility of "cluster-associated" study design effects, two sensitivity analyses will be considered. First, the main analysis will be repeated using the physician as the unit of analysis. Second, the aforementioned logistic regression models will be reanalyzed using generalized estimating equations to control for the potential lack of statistical independence among patients treated by the same study physician[84].

There will be one pre-planned interim analysis to explore event rates in all three study arms after 80 patients per arm have reached the six month primary outcome time-point. We are primarily interested in examining whether projected event rates are correct, or whether the sample size will need to be adjusted upwards, and will employ the Haybittle-Peto stopping rule using a Z value of 3.0 for this interim test. For the main study analysis, we will consider a p-value < 0.05 to be statistically significant.

#### Other analyses

In examining the secondary outcomes (for example, use of ACE inhibitors, beta-blockers, etc.), we will use similar statistical methods for the primary statin outcome analyses.

### Data management

All data will be collected using standardized data sheets and data collation, entry and quality assurance will be carried out in the Epidemiology Coordinating and Research (EPICORE) Centre, Division of Cardiology, University of Alberta.

### Ethical considerations

Each patient will be given written information about the study and written informed consent will be obtained prior to study entry. Although the physicians receiving our study faxes will not be explicitly informed that they are in a trial at the time of the fax, we did inform all physicians with privileges within participating health authorities at the time of the opinion leader survey that a trial testing novel strategies to communicate evidence for patients with coronary artery disease would be conducted in the near future. On the survey form eliciting the opinion leaders, we gave all physicians the option to indicate if they did not want to participate in this future region-wide program to improve prescribing practices for CAD. Those who declared they did not want to participate were excluded from ESP-CAD eligibility. Thus, any patients of these physicians will not be enrolled in ESP-CAD. Furthermore, it should be noted that as part of the process of obtaining privileges within the participating health authorities (the Capital Health Authority in Edmonton and the Calgary Health Region) physicians sign a consent form to participate in "peer review and clinical quality improvement programs" within the health authorities, and such forms are updated every three years.

The study protocol has been approved by the Health Research Ethics Board, University of Alberta, Edmonton, Alberta (file number: 5082) and the Conjoint Health Research Ethics Board, University of Calgary, Calgary, Alberta (ethics ID: E-20129). The funding for the study is from three peer-reviewed grants. The funding sources (the Alberta Heritage Foundation for Medical Research, the Heart and Stroke Foundation of Canada, and Pfizer Canada) had no role in the design of the study and will have no role in its conduct, analysis, interpretation, or reporting – and will not have access to the data. None of the local opinion leaders received any financial compensation for their participation in the study or endorsement of the evidence summaries.

## Discussion

We report the protocol of a cluster randomized trial that aims to determine the effect of a feasible and readily generalizable evidence-based quality improvement initiative for patients with CAD. Local opinion leaders are potentially powerful tools for quality improvement, and our proposed method of defining a role for them in cardiovascular disease should lead to improved care for patients. In addition, this trial will establish whether the APPROACH computer system can be used as a novel vehicle for identifying and delivering secondary prevention interventions to high-risk patients with CAD. If our interventions are effective, they can be widely and easily applied in other communities, particularly in the future as APPROACH extends beyond the borders of Alberta, as well as for other target conditions.

## Competing interests

The author(s) declare that they have no competing interests.

## Authors' contributions

FM conceived and designed the study with input from all authors. FM and MF drafted this manuscript, although all authors provided comments on the drafts and have read and approved the final version.

## Supplementary Material

Additional File 1 The Opinion Leader Statement. Click here for file

Additional File 2 The Unsigned Evidence Statement. Click here for file

## References

[B1] Padwal R, Straus SE, McAlister FA (2001). Cardiovascular risk factors and their effects on the decision to treat hypertension: evidence based review. BMJ.

[B2] 4S Investigators (1994). Randomised trial of cholesterol lowering in 4444 patients with coronary heart disease: the Scandinavian Simvastatin Survival Study (4S). Lancet.

[B3] The Long-Term Intervention with Pravastatin in Ischaemic Disease (LIPID) Study Group (1998). Prevention of cardiovascular events and death with pravastatin in patients with coronary heart disease and a broad range of initial cholesterol levels. N Engl J Med.

[B4] Sacks FM, Pfeffer MA, Moye LA, Rouleau JL, Rutherford JD, Cole TG, Brown L, Warnica JW, Arnold MO, Wun C, Davis BR, Braunwald E (1996). The effect of pravastatin on coronary events after myocardial infarction in patients with average cholesterol levels. N Engl J Med.

[B5] Heart Protection Study Collaborative Group (2002). MRC/BHF Heart Protection Study of cholesterol lowering with simvastatin in 20 536 high-risk individuals: a randomised placebo-controlled trial. Lancet.

[B6] Schwartz GG, Olsson AG, Ezekowitz MD, Ganz P, Oliver MF, Waters D, Zeiher A, Chaitman BR, Leslie S, Stern T (2001). Effects of atorvastatin on early recurrent ischemic events in acute coronary syndromes: the MIRACL study: a randomized controlled trial. JAMA.

[B7] Serruys PWJC, de Feyter P, Macaya C, Kobott N, Puel J, Vrolix M, Branzi A, Bertolami MC, Jackson G, Strauss B, Meier B (2002). Fluvastatin for prevention of cardiac events following successful first percutaneous coronary intervention. A randomized controlled trial. JAMA.

[B8] Shepherd J, Blauw GJ, Murphy MB, Bollen EL, Buckely BM, Cobbe SM, Ford P, Gaw A, Hyland M, Jukema JW, Kamper AM, Macfarlane W, Meinders E, Norrie J, Packard CJ, Perry PJ, Stott DJ, Sweeney BJ, Twomey G, Westendorp GJ (2002). Pravastatin in elderly individuals at risk of vascular disease (PROSPER): a randomised controlled trial. Lancet.

[B9] Sever PS, Dahlof B, Poulter NR, Wedel H, Beevers G, Caulfiel M, Collins R, Kjeldsen SE, Kristinsson A, McInnes GT, Mehlsen J, Nieminen M, O'Brien E, Ostergren J (2003). Prevention of coronary and stroke events with atorvastatin in hypertensive patients who have average or lower-than-average cholesterol concentrations, in the Anglo-Scandinavian Cardiac Outcomes Trial- Lipid Lowering Arm (ASCOT-LLA): a multicentre randomised controlled trial. Lancet.

[B10] Cannon CP, Braunwald E, McCabe CH, Rader DJ, Rouleau JL, Belder R, Joyal SV, Hill KA, Pfeffer MA, Skene AM (2004). Intensive versus moderate lipid lowering with statins after acute coronary syndromes. N Engl J Med.

[B11] LaRosa JC, Grundy SM, Waters DD, Shear C, Barter P, Fruchart JC, Gotto AM, Greten H, Kastelein JJP, Shepard J, Wenger NK (2005). Intensive lipid lowering with atorvastatin in patients with stable coronary disease. N Engl J Med.

[B12] Pederen TR, Faergeman O, Kastelein JJP, Olsson AG, Tikkanen MJ, Holme I, Larsen ML, Bendiksen FS, Lindahl C, Szarek M, Tsai J (2005). High-dose atorvastatin vs usual-dose simvastatin for secondary prevention after myocardial infarction. JAMA.

[B13] Shepherd J, Cobbe SM, Ford I, Isles CG, Lorimer AR, MacFarlane PW, McKillop JH, Packard CJ (1995). Prevention of coronary heart disease with pravastatin in men with hypercholesterolemia. West of Scotland Coronary Prevention Study Group. N Engl J Med.

[B14] Downs JR, Clearfield M, Weis S, Whitney E, Shapiro DR, Beere PA, Langendorfer A, Stein EA, Kruer W, Gotto AM (1998). Primary prevention of acute coronary events with lovastatin in men and women with average cholesterol levels: results of AFCAPS/TexCAPS. Air Force/Texas Coronary Atherosclerosis Prevention Study. JAMA.

[B15] The ALLHAT Officers and Coordinators for the ALLHAT Collaborative Research Group (2002). Major outcomes in moderately hypercholesterolemic, hypertensive patients randomized to pravastatin vs. usual care (ALLHAT-LLT). JAMA.

[B16] Cholesterol Treatment Trialists' (CTT) Collaborators (2005). Efficacy and safety of cholesterol-lowering treatment: prospective meta-analysis of data from 90 056 participants in 14 randomised trials of statins. Lancet.

[B17] Pechacek TF, Asma S, Eriksen MP, Yusuf S, Cairns A, Camm AJ, Fallen EL, Gersch BJ (1998). Tobacco: global burden and community solutions. Evidence Based Cardiology.

[B18] Law M, Tang JL (1995). An analysis of the effectiveness of interventions intended to help people stop smoking. Arch Intern Med.

[B19] Lancaster T, Stead LF (2001). Individual behavioural counselling for smoking cessation. The Cochrane Library.

[B20] Ashenden R, Silagy C, Weller D (1997). A systematic review of the effectiveness of promoting lifestyle change in general practice. Family Practice.

[B21] Taylor CB, Houston-Miller N, Killen JD, DeBusk RF (1990). Smoking cessation after acute myocardial infarction: effects of a nurse-managed intervention. Ann Intern Med.

[B22] Antithrombotic Trialists Collaboration (2002). Collaborative meta-analysis of randomised trials of antiplatelet therapy for prevention of death, myocardial infarction, and stroke in high risk patients. BMJ.

[B23] Yusuf S, Peto R, Lewis J, Collins R, Sleight P (1985). Beta blockade during and after myocardial infarction: an overview of the randomised trials. Prog Cardiovasc Dis.

[B24] The Beta-Blocker Pooling Project Research Group (1988). The Beta-Blocker Pooling Project (BBPP): subgroup findings from randomized trials in post-infarction patients. Eur Heart J.

[B25] Heidenreich PA, McDonald KM, Hastie T, Fadel B, Hagan V, Lee BK, Hlatky MA (1999). Meta-analysis of trials comparing beta-blockers, calcium antagonists, and nitrates for stable angina. JAMA.

[B26] Rehnqvist N, Hjemdahl P, Billing E, Bjorkander I, Eriksson SV, Forslund L, Held C, Nasman P, Wallen HN (1995). Treatment of stable angina pectoris with calcium antagonists and beta-blockers. The APSIS (Angina Prognosis Study in Stockholm) Study. Cardiologia.

[B27] Pepine CJ, Cohn PF, Deedwania PC, Gibson RS, Handberg E, Hill JA, Miller E, Marks RG, Thadani U (1994). Effects of treatment on outcome in mildly symptomatic patients with ischemia during daily life. The Atenolol Silent Ischemia Study (ASIST). Circulation.

[B28] Dargie HJ, Ford I, Fox KM (1996). Total Ischaemic Burden European Trial (TIBET). Effects of ischaemia and treatment with atenolol, nifedipine SR, and their combination on outcomes in patients with chronic stable angina. The TIBET Study Group. Eur Heart J.

[B29] Savonitto S, Ardissiono D, Egstrup K, Rasmussen K, Bae EA, Omland T, Schjelderup-mathiesen PM, Marraccini P, Wahlqvist I, Merlini PA, Rehnqvist N (1996). Combination therapy with metoprolol and nifedipine versus monotherapy in patients with stable angina pectoris. Results of the International Multicenter Angina Exercise (IMAGE) Study. J Am Coll Cardiol.

[B30] De Vries RJ, van den Heuvel AF, Lok DJ, Claessens RJ, Bernink PJ, Pasteuninge WH, Kingma JH, Sunselman PH (1996). Nifedipine gastrointestinal therapeutic system versus atenolol in stable angina pectoris. Int J Cardiol.

[B31] Von Arnim T (1995). Medical treatment to reduce total ischemic burden: total ischemic burden bisoprolol study (TIBBS), a multicenter trial comparing bisoprolol and nifedipine. J Am Coll Cardiol.

[B32] Fihn SD, Williams SV, Daley J, Gibbons RJ (2001). Guidelines for the management of patients with chronic stable angina: treatment. Ann Intern Med.

[B33] Heart Outcomes Prevention Evaluation Study Investigators (2000). Effects of an angiotensin-converting-enzyme inhibitor, ramipril, on cardiovascular events in high-risk patients. N Engl J Med.

[B34] The EURopean trial On reduction of cardiac events with Perindopril in stable coronary Artery disease Investigators (2003). Efficacy of perindopril in reduction of cardiovascular events among patients with stable coronary artery disease: randomized, double-blind, placebo-controlled, multicentre trial (the EUROPA study). Lancet.

[B35] The Simvastatin/Enalapril Coronary Atherosclerosis Trial (SCAT) (2000). Long-term effects of cholesterol lowering and angiotensin-converting enzyme inhibition on coronary atherosclerosis. Circulation.

[B36] The PEACE Trial Investigators (2004). Angiotensin-converting-enzyme inhibition in stable coronary artery disease. N Engl J Med.

[B37] McCormick D, Gurwitz JH, Lessard D, Yarzebski J, Gore JM, Goldberg RJ (1999). Use of aspirin, beta-blockers, and lipid-lowering medications before recurrent acute myocardial infarction: missed opportunities for prevention?. Arch Intern Med.

[B38] Majumdar SR, Gurwitz JH, Soumerai SB (1999). Undertreatment of hyperlipidemia in the secondary prevention of coronary artery disease. J Gen Intern Med.

[B39] Gottlieb SS, McCarter RJ, Vogel RA (1998). Effect of beta-blockade on mortality among high-risk and low-risk patients after myocardial infarction. N Engl J Med.

[B40] EUROASPIRE I and II Group (2001). Clinical reality of coronary prevention guidelines: a comparison of EUROASPIRE I and II in nine countries. Lancet.

[B41] Campbell NC, Thain J, Dean HG, Ritchie LD, Rawles JM (1998). Secondary prevention in coronary heart disease: baseline survey of provision in general practice. BMJ.

[B42] Xhignesse M, Laplante P, Grant AM, Niyonsenga T, Delisle E, Vanasse N, Bernier R (1999). Antiplatelet and lipid-lowering therapies for the secondary prevention of cardiovascular disease: are we doing enough?. Can J Cardiol.

[B43] Rojas-Fernandez CH, Kephart GC, Sketris IS, Kass K (1999). Underuse of acetylsalicylic acid in individuals with myocardial infarction, ischemic heart disease or stroke: data from the 1995 population-based Nova Scotia Health Survey. Can J Cardiol.

[B44] Brady AJB, Oliver MA, Pittard JB (2001). Secondary prevention in24 431 patients with coronary heart disease: survey in primary care. BMJ.

[B45] Barron HV, Michaels AD, Maynard C, Every NR (1998). Use of angiotensin-converting enzyme inhibitors at discharge in patients with acute myocardial infarction in the United States: data from the National Registry of Myocardial Infarction 2. J Am Coll Cardiol.

[B46] Wang TJ, Stafford RS (1998). National patterns and predictors of beta-blocker use in patients with coronary artery disease. Arch Intern Med.

[B47] Califf RM, DeLong ER, Ostbye T, Muhlbaier LH, Chen A, LaPointe NA, Hammill BG, McCants CB, Kramer JM (2002). Underuse of aspirin in a referral population with documented coronary artery disease. Am J Cardiol.

[B48] Pilote L, Beck CA, Karp I, Alter D, Austin P, Cox J, Humphries K, Jackevicius C, Richard H, Tu JV (2004). Secondary prevention after acute myocardial infarction in four Canadian provinces, 1997–2000. Can J Cardiol.

[B49] Bourgault C, Davignon J, Fodor G, Gagne C, Gaudet D, Genest J, Lavoie MA, Leiter L, McPherson R, Senecal M, Marentette M, Sebaldt (2005). Statin therapy in Canadian patients with hypercholesterolemia: the Canadian lipid study-observational (CALIPSO). Can J Cardiol.

[B50] Bhatt DL, Steg PG, Ohman EM, Hirsch AT, Ikeda Y, Mas JL, Goto S, Liau CS, Richard AJ, Rother J, Wilson PWF (2006). International prevalence, recognition, and treatment of cardiovascular risk factors in outpatients with atherothrombosis. JAMA.

[B51] Jackevicius CA, Mamdani M, Tu JV (2002). Adherence with statin therapy in elderly patients with and without acute coronary syndromes. JAMA.

[B52] Benner JS, Glynn RJ, Mogun H, Neumann PJ, Weinstein MC, Avron J (2002). Long-term persistence in use of statin therapy in elderly patients. JAMA.

[B53] Newby LK, Allen LaPointe NM, Chen AY, Kramer JM, Hammill BG, DeLong ER, Muhlbaier LH, Califf RM (2006). Long-term adherence to evidence-based secondary prevention therapies in coronary artery disease. Circulation.

[B54] Mehta RH, Montoye CK, Gallogly M, Baker P, Blount A, Faul J, Roychoudhury C, Borzak S, Fox S, Franlkin M, Freundl M, Kline-Rogers E, LaLonde T, Orza M, Parrish R, Satwicz M, Smith MJ, Sobotka P, Winston S, Riba AA, Eagle KA (2002). Improving quality of care for acute myocardial infarction. The Guidelines Applied in Practice (GAP) Initiative. JAMA.

[B55] Davis DA, Thomson MA, Oxman AD, Haynes RB (1995). Changing physician performance. A systematic review of the effect of continuing medical education strategies. JAMA.

[B56] Oxman AD, Thomson MA, Davis DA, Haynes RB (1995). No magic bullets: a systematic review of 102 trials of interventions to improve professional practice. CMAJ.

[B57] Clark AM, Hartling L, Vandermeer B, McAlister FA (2005). Randomized trials of secondary prevention programs in coronary heart disease: a systematic review. Ann Intern Med.

[B58] Majumdar SR, McAlister FA, Furberg CD (2004). From publication to practice in chronic cardiovascular disease- the long and winding road. J Am Coll Cardiol.

[B59] Grimshaw JM, Shirran L, Thomas R, Mowatt G, Fraser C, Bero L, Grilli R, Harvey E, Oxman A, O'Brien MA (2001). Changing provider behavior. An overview of systematic reviews of interventions. Med Care.

[B60] Hayward RS, Guyatt GH, Moore KA, McKibbon KA, Carter AO (1997). Canadian physicians' attitudes about and preferences regarding clinical practice guidelines. CMAJ.

[B61] McAlister FA, Graham I, Karr GW, Laupacis A (1999). Evidence-based medicine and the practicing clinician. J Gen Intern Med.

[B62] Feder G, Griffiths C, Eldridge S, Spence M (1999). Effect of postal prompts to patients and general practitioners on the quality of primary care after a coronary event (POST): randomised controlled trial. BMJ.

[B63] Derose SF, Dudl JR, Benson VM, Contreras R, Nakahiro RK, Ziel FH (2005). Point of service reminders for prescribing cardiovascular medications. Am J Managed Care.

[B64] Eccles M, McColl E, Steen N, Rousseau N, Grimshaw J, Parkin D, Purves I (2002). Effect of computerized evidence based guidelines on management of asthma and angina in adults in primary care: cluster randomized controlled trial. BMJ.

[B65] Tierney WM, Overhage JM, Murray MD, Harris LE, Zhou XH, Eckert GJ, Smith FE, Nienaber N, McDonald CJ, Wolinsky FD (2003). Effects of computerized guidelines for managing heart disease in primary care. J Gen Intern Med.

[B66] Sequist TD, Gandhi TK, Karson AS, Fiskio JM, Bugbee D, Sperling M, Cook EF, Orav EJ, Fairchild DB, Bates DW (2005). A randomized trial of electronic clinical reminders to improve quality of care for diabetes and coronary artery disease. J Am Med Inform Assoc.

[B67] Lester WT, Grant RW, Barnett GO, Chueh HC (2006). Randomized controlled trial of an informatics-based intervention to increase statin prescription for secondary prevention of coronary disease. J Gen Intern Med.

[B68] Hiss RG, MacDonald R, David WR (1978). Identification of physician educational influentials in small community hospitals. Res Med Educ.

[B69] Thomson O'Brien MA, Oxman AD, Haynes RB, Davis DA, Freemantle N, Harvey EL (2002). Local opinion leaders: effects on professional practice and health care outcomes (Cochrane Review). The Cochrane Library.

[B70] Majumdar SR, Lipton HL, Soumerai SB, Strom B (2005). Chapter 28 – Evaluating and improving physician prescribing. ed Pharmacoepidemiology.

[B71] Majumdar SR, Tsuyuki RT, McAlister FA Impact of opinionleader endorsed evidence on quality of prescribing for patients withcardiovascular disease: randomized controlled trial[ISRCTN26365328].

[B72] Berner ES, Baker CS, Funkhouser E, Heudebert GR, Allison JJ, Fargason CA, Li Q, Person SD, Kiefe CI (2003). Do local opinion leaders augment hospital quality improvement efforts? A randomized trial to promote adherence to unstable angina guidelines. Medical Care.

[B73] Lomas J, Enkin M, Anderson GM, Hannah WJ, Vayda E, Singer J (1991). Opinion leaders vs audit and feedback to implement practice guidelines. Delivery after previous cesarean section. JAMA.

[B74] Soumerai SB, McLaughlin TJ, Gurwitz JH, Guadagnoli E, Hauptman PJ, Borbas C, Morris N, McLaughlin B, Gao X, Willison DJ, Asinger R, Gobel F (1998). Effect of local medical opinion leaders on quality of care for acute myocardial infarction: a randomized controlled trial. JAMA.

[B75] Hayward RS, Wilson MC, Tunis SR, Guyatt GH, Moore KA, Bass EB (1996). Practice guidelines. What are internists looking for ?. J Gen Intern Med.

[B76] Shekelle PG, Kravitz RL, Beart J, Marger M, Wang M, Lee M (2000). Are nonspecific practice guidelines potentially harmful? A randomized comparison of the effect of nonspecific versus specific guidelines on physician decision making. Health Services Research.

[B77] Grol RJ, Dalhuijsen S, Thomas C, in't Veld C, Rutten G, Mokkink H (1998). Attributes of clinical practice guidelines that influence the use of guidelines in general practice: observational study. BMJ.

[B78] Majumdar SR, McAlister FA, Tsuyuki RT (2005). A cluster randomized trial to assess the impact of opinion leader endorsed evidence summaries on improving quality of prescribing for patients with chronic cardiovascular disease: rational and design [ISRCTN26365328]. BMC Cardiovascular Disorders.

[B79] Underwood M, Barnett A, Hajioff S (1998). Cluster randomization:a trap for the unwary. Br J Gen Practice.

[B80] Eccles M, Grimshaw J, Campbell M, Ramsay C (2003). Researchdesigns for studies evaluating the effectiveness of change and improvement strategies. Quality and Safety in Health Care.

[B81] Ghali WA, Knudtson ML, on behalf of the APPROACH investigators (2000). Overview of the Alberta Provincial Project for Outcome Assessment in Coronary Heart Disease. Can J Cardiol.

[B82] Graham MM, Faris PD, Ghali WA, Galbraith PD, Norris CM, Badry JT, Mitchell LB, Curtis MJ, Knudtson ML (2001). Validation of three myocardial jeopardy scores in a population-based cardiac catheterization cohort. Am Heart J.

[B83] Klinke JA, Johnson JA, Guirguis LM, Toth EL, Lee TK, Lewanzcuk RZ, Majumdar SR (2004). Underuse of aspirin in type-2 diabetes mellitus:prevalence and correlates of therapy in rural Canada. Clin The rapeutics.

[B84] Diggle PJ, Liang KY, Zeger SL (1996). Analysis of longitudinal data.

