# Supplementary material for: A randomized trial to assess the impact of opinion leader endorsed evidence summaries on the use of secondary prevention strategies in patients with coronary artery disease: the ESP-CAD trial protocol [NCT00175240]
Source: Implement Sci. 2006 May 6;1:11. doi: 10.1186/1748-5908-1-11 (PMC1475885; doi:10.1186/1748-5908-1-11)
Supplement: Additional File 2 — The Unsigned Evidence Statement. [file 1748-5908-1-11-S2.pdf]

Unsigned Evidence Statement for Coronary Artery Disease

-date-

Dr. xxxx
FAX:

Dear Dr. xxxx,

Based on the attached coronary angiogram, your patient _(insert pt name)__ has **coronary artery disease** and therefore has more than a 30% 10-year risk of a cardiovascular event. As you know, patients like this derive significant benefit from many medications (assuming they don't have contraindications).

- **Statins** consistently reduce mortality (by 25% to 30%) and morbidity, including the need for revascularization, in patients with **any degree** of coronary artery disease or **any degree** of dyslipidemia. Optimal benefit from statin therapy is achieved if the LDL can be lowered to below 2.5 mmol/L (or possibly even 1.8 mmol/L in some patients), but even patients with "normal" cholesterols appear to benefit from statins if they have coronary artery disease.
- **ACE inhibitors** reduce mortality by 25% in patients with heart failure and/or coronary artery disease.
- **Antiplatelet agents** (such as aspirin) reduce myocardial infarcts and strokes by 23% in patients with coronary disease.
- **Beta-blockers** reduce mortality by 25% in myocardial infarct survivors, and should be used for treatment of angina or hypertension in patients with coronary disease.
- **Smoking cessation** in smokers with coronary disease reduces their MI risk by 50% within 2 years. Even a single recommendation by a physician can increase quitting rates by 2%.

Therefore, the following approach is recommended for patients like_(insert pt name)__:

1. A fasting lipid profile (if not done in the last year) and **starting a statin** of your choice (for example: simvastatin 20 mg daily increasing to 80 mg daily if necessary, pravastatin 10 mg daily increasing to 40 mg daily if necessary, atorvastatin 10 mg daily increasing to 80 mg daily if necessary, or fluvastatin 20 mg daily increasing to 80 mg daily if necessary). Statins are contraindicated in patients with cirrhosis, elevated ALT or AST, inflammatory muscle disease, women who are trying to get pregnant, and should be used with caution if creatinine exceeds 200 umol/L.

2. **Follow-up tests in 6 weeks**, including a repeat fasting lipid profile, CK, and liver function tests (ALT or AST).

3. **For patients on a statin, the dose should be titrated up every 6 weeks (if necessary) to achieve an LDL level below 2.5 mmol/L.** CK and liver function tests (ALT or AST) should be monitored 6 weeks after dosage changes and intermittently in patients on stable dose.

4. In smokers, **smoking cessation** should be advised (including the use of nicotine replacement products or Zyban in selected cases).

5. In those without contraindications, **ACE inhibitors** and **aspirin** should be considered. Patients started on ACE inhibitors should have their electrolytes and creatinine monitored within 1-2 weeks and intermittently thereafter.

6. In those with prior MI or angina symptoms and without contraindications, **beta-blockers** should be considered.

These recommendations are based on recently published studies and guidelines. If you have any questions or concerns, or would like any of the literature supporting these recommendations, please contact our knowledge translation assistant, John Doe (phone: xxx-xxxx; fax: xxx-xxxx).

In addition, for those interested in CME credits, you may collect 2 MAINPRO M2 credits from the College of Family Physicians of Canada by initialing a copy of this page and keeping it in your CME records.
